# Children with Early-Onset Psychosis Have Increased Burden of Rare *GRIN2A* Variants

**DOI:** 10.3390/genes14040779

**Published:** 2023-03-23

**Authors:** Margaret A. Hojlo, Merhawi Ghebrelul, Casie A. Genetti, Richard Smith, Shira Rockowitz, Emma Deaso, Alan H. Beggs, Pankaj B. Agrawal, David C. Glahn, Joseph Gonzalez-Heydrich, Catherine A. Brownstein

**Affiliations:** 1 Early Psychosis Investigation Center (EPICenter), Boston Children’s Hospital, Boston, MA 02115, USA; 2 Tommy Fuss Center for Neuropsychiatric Disease Research, Boston Children’s Hospital, Boston, MA 02115, USA; 3 Department of Psychiatry and Behavioral Sciences, Boston Children’s Hospital, Boston, MA 02115, USA; 4 The Manton Center for Orphan Disease Research, Boston Children’s Hospital, Boston, MA 02115, USA; 5 Division of Genetics and Genomics, Boston Children’s Hospital, Boston, MA 02115, USA; 6 Department of Pharmacology, Feinberg School of Medicine, Northwestern University, Chicago, IL 60611, USA; 7 Research Computing, Information Technology, Boston Children’s Hospital, Boston, MA 02115, USA; 8 Department of Pediatrics, Harvard Medical School, Boston, MA 02115, USA; 9 Division of Neonatology, Department of Pediatrics, University of Miami Miller School of Medicine, Holtz Children’s Hospital, Jackson Health System, Miami, FL 33136, USA; 10 Department of Psychiatry, Harvard Medical School, Boston, MA 02115, USA

**Keywords:** psychosis, genetics, genomics, schizophrenia

## Abstract

Background: Children and adolescents with early-onset psychosis (EOP) have more rare genetic variants than individuals with adult-onset forms of the illness, implying that fewer EOP participants are needed for genetic discovery. The Schizophrenia Exome Sequencing Meta-analysis (SCHEMA) study predicted that 10 genes with ultra-rare variation were linked to adult-onset schizophrenia. We hypothesized that rare variants predicted “High” and “Moderate” by the Variant Effect Predictor Algorithm (abbreviated as VEPHMI) in these 10 genes would be enriched in our EOP cohort. Methods: We compared rare VEPHMI variants in individuals with EOP (N = 34) with race- and sex-matched controls (N = 34) using the sequence kernel association test (SKAT). Results: *GRIN2A* variants were significantly increased in the EOP cohort (*p* = 0.004), with seven individuals (20% of the EOP cohort) carrying a rare VEPHMI variant. The EOP cohort was then compared to three additional control cohorts. *GRIN2A* variants were significantly increased in the EOP cohort for two of the additional control sets (*p* = 0.02 and *p* = 0.02), and trending towards significance for the third (*p* = 0.06). Conclusion: Despite a small sample size, *GRIN2A* VEPHMI variant burden was increased in a cohort of individuals with EOP in comparison to controls. *GRIN2A* variants have been associated with a range of neuropsychiatric disorders including adult-onset psychotic spectrum disorder and childhood-onset schizophrenia. This study supports the role of *GRIN2A* in EOP and emphasizes its role in neuropsychiatric disorders.

## 1. Introduction

Schizophrenia is a complex neurodevelopmental condition with a lifetime prevalence estimated to be just under 1% worldwide [1,2,3]. Schizophrenia symptoms include a combination of positive symptoms such as hallucinations, delusions, and disorganized behavior; negative symptoms such as social withdrawal and diminished speech; and cognitive impairments [1]. Symptoms typically emerge in late adolescence and early adulthood, but early-onset and childhood-onset cases, while much more rare, also occur. Individuals with early-onset and childhood-onset schizophrenia experience psychotic symptoms before ages 18 and 13 years, respectively [4]. Childhood-onset schizophrenia is considered to be an especially severe form of psychosis, with a higher rate of brain abnormalities as well as greater environmental and genetic risk factors [5,6].

Individuals with childhood-onset schizophrenia (COS) have a nearly three-fold increase of recurrent genomic copy number variations (CNVs) relative to those with adult-onset schizophrenia [6,7]. This disparity suggests an increased contribution of rare genetic variation to the etiopathology of childhood-onset schizophrenia. However, fewer than half of children and adolescents with a psychotic spectrum disorder meet the strict criteria for schizophrenia [8] and childhood psychiatric diagnoses often change over the course of development [9,10]. Consequently, there is considerable interest in understanding the genetic underpinnings of the more inclusive early-onset psychosis (EOP) categorization, which is defined as any psychiatric diagnosis with pronounced psychotic symptoms that is onset prior to 18 years of age [11]. The EOP categorization captures psychotic symptomatology across the spectrum of diagnostic criteria and is therefore more representative of the phenotypic variability in children and adolescents with psychotic disorders. Existing research has demonstrated that, compared to adult-onset illness, EOP may be associated with lower cognitive and psychosocial functioning, longer duration of untreated illness, more hospitalizations, and less functional improvement over time [8,12].

We recently documented that similarly to childhood-onset schizophrenia, children and adolescents with the broader EOP diagnosis have a two- to three-fold increase in recurrent CNVs relative to individuals with adult-onset schizophrenia [13]. This suggests that genetic burden, particularly for rare high penetrant mutations, is higher for individuals with EOP than it is for those with typical adult-onset psychotic spectrum disorders. Thus, EOP cohorts that are much smaller than those required to study adult-onset schizophrenia can provide important insights into the rare genetic underpinnings of psychosis. 

The recent report of the Schizophrenia Exome Sequencing Meta-analysis (SCHEMA) consortia [14] represents a major advance in our understanding of the genetic architecture of schizophrenia. Through meta-analysis of whole-exomes from 24,248 cases and 97,322 controls, the SCHEMA consortia reported that ultra-rare coding variants in 10 genes (*TRIO*, *CUL1*, *XPO7*, *SP4*, *SETD1A*, *CACNA1G*, *GRIA3*, *HERC1*, *RB1CC1*, and *GRIN2A*) confer substantial risk for schizophrenia (odds ratios 3–50, *p* < 2.14 × 10^−6^) [14]. Many of the genes included in the SCHEMA consortia list have also emerged as strong candidates for other neuropsychiatric conditions, such as bipolar disorder, major depressive disorder, and autism spectrum disorder [15,16,17,18,19,20,21,22], indicating that these genes have a broad continuum of influence on psychosis spectrum, mood, and neurodevelopmental disorders. The findings of the SCHEMA study have exciting potential to significantly impact the development of targeted therapies for schizophrenia and related disorders, as well as to enhance our understanding of the underlying biological mechanisms of these conditions.

Despite the importance of this herculean effort, the observation of *SCHEMA* genes in an independent sample has yet to be replicated. Early-onset and severe phenotypes often have a higher genetic component than later-onset or milder forms of disorders, and therefore provide a more efficient route for identifying genetic factors [23,24,25]. Identifying genetic factors involved in early-onset and severe forms of a disease can also provide important insights into the underlying biological mechanisms of the disorder. Therefore, we would expect the 10 genes named by *SCHEMA* to have rare and damaging variation that is overrepresented in early-onset presentations, and for this overrepresentation to be an important validation of the genes’ involvement in psychosis spectrum disorders. The goal of this study was to attempt such a SCHEMA replication in a cohort of individuals with EOP (N = 34) and matched unaffected controls (N = 34). More specifically, we tested the hypothesis that the burden of rare (MAF < 0.05) variants predicted to be of “High” or “Moderate” impact on the Variant Effect Predictor Algorithm [26] in the 10 *SCHEMA* genes would be enriched in the EOP group relative to the controls.

## 2. Materials and Methods

EOP Samples: Unrelated children, adolescents, and young adults with EOP were referred to the Developmental Neuropsychiatry Program in the Department of Psychiatry at Boston Children’s Hospital. Prior to enrollment into the cohort, clinical diagnoses were ascertained by a board-certified child psychiatrist (JGH) who specializes in EOP. Diagnoses were subsequently confirmed via medical record review with a Diagnostic and Statistical Manual of Mental Disorders (Fifth Edition) (DSM-5) checklist. Age of onset of psychotic symptoms and demographic information (sex, race, and ethnicity) were also collected from the medical record. Ethnicity categories reflect preset options in the medical record. 

Inclusion criteria were having a DSM-5 diagnosis for current or lifetime Axis I schizophrenia spectrum or other psychotic disorder with onset prior to age 18. Exclusion criteria were (1) substance- or medication-induced psychosis; (2) psychosis secondary to a brain infection (e.g., encephalitis); and (3) psychosis due to a neurodegenerative disorder such as Wilson’s disease, Dystonia muscular deformations, Huntington’s disease, Friedrich’s ataxia, Ataxia Telangiectasia, or Parkinson’s disorder. Diagnoses included schizophrenia, schizoaffective disorders, and unspecified and other specified schizophrenia spectrum and other psychotic disorders. 

All EOP participants or caregivers/legal guardians provided written informed consent (and participants <18 years provided assent when possible) on forms approved by the Boston Children’s Hospital Institutional Review Board as part of the Manton Center for Orphan Disease Research protocol. After providing written informed consent/assent, each participant provided peripheral blood samples for DNA extraction. 

Control Samples: Comparison samples consisted of sex- and racially-matched unaffected biological parents of individuals with suspected Mendelian disorders who are enrolled in the Manton Center for Orphan Disease Research protocol. 

Sequencing: Whole exome sequencing on Illumina short read platforms for the EOP cohort was conducted by the Yale Center for Mendelian Genomics (New Haven, CT, USA) and controls were sequenced through the Broad Institute Center for Mendelian Genomics (Cambridge, MA, USA). Analysis was performed using Codified Genomics (San Diego, CA, USA) and Genuity Science (Dublin, Ireland). Variants in the analysis included those predicted “Moderate” or “High” impact by Ensembl’s Variant Effect Predictor Algorithm (abbreviated as VEPHMI) and include transcript ablation, splice acceptor variant, splice donor variant, stop gained, frameshift variant, stop lost, start lost, transcript amplification, inframe insertion, inframe deletion, missense variant, protein altering variant, splice region variant, and/or incomplete terminal codon variant [26].

Data Processing: We created a “freeze”, which is a proprietary data model developed by Genuity Science that is similar to a joint-called variant call format, or VCF, within the Genuity Science platform [27]. Recognizing that the cases and controls were sequenced at different sequencing facilities, we were particularly concerned to make sure that the coverage within the 10 genes of interest was robust. The overall coverage of the sequencing reads for Variant Effect Predictor (VEP) High or Moderate variants across each of the cases and controls was high, despite the mixed sequencing facilities, with more than 94% of the VEP High or Moderate variants included in the freeze being assigned to either homozygous reference, heterozygous call, or homozygous call in each individual. This 94% quality metric for the freeze ensured the validity of comparing the two cohorts. 

A set of 138 housekeeping genes (see Supplementary Table S1) was used as a control to ensure that there were no systematic differences between case and control sequences (e.g., increased rare variant calls in the EOP cohort). The housekeeping genes were selected by a pseudorandom number generator from a list of 3803 housekeeping genes from Eisenberg et al. [28]. 

Statistical Methods: The sequence kernel association test (SKAT) was used to test for group differences in VEPHMI burden between the EOP and control samples for each of the 10 *SCHEMA* genes separately. SKAT is a supervised, flexible, computationally efficient regression method to test for association between genetic variants (common and rare) in a region and a continuous or dichotomous trait [29]. SKAT analysis [30] was performed in Genuity Science (CSA: 5.16.2, Sequence miner: 13.2.9, GOR: 12.6.2) [31] using GRCh37. Allele frequency upper threshold was 0.05, with a Harvey-Weinberg equilibrium threshold of 1e-6. Bonferroni correction for the 10 genes tested adjusted the α level to 0.005.

Additional Control Analyses: After a gene with an increased incidence in the EOP cohort was identified, three additional race-matched control cohorts were created to further verify the finding. Each additional control cohort consisted of unaffected biological parents of individuals with suspected Mendelian disorders who were enrolled in the Manton Center for Orphan Disease Research protocol. These additional control cohorts were also compared to the EOP cohort using SKAT. 

## 3. Results

EOP Participants: The EOP cohort included 34 participants (38.2% female). EOP participants were African American/African (17.6%), Asian or Pacific Islander (2.9%), European American/European (61.8%), and Other (2.9%). Five participants (14.7%) declined to answer/race was unknown. About one fifth of participants were Hispanic/Latino (20.6%). The average age of onset of psychotic symptoms was 9.6 years (SD: 3.1) and co-occurring psychiatric and neurodevelopmental diagnoses, such as attention-deficit/hyperactivity disorder (ADHD), were common. Demographics of the EOP cohort are outlined in Table 1. 

Genetic Burden: Using SKAT, VEPHMI rare variant burden was first compared between individuals with EOP (N = 34) and an initial set of controls (N = 34) (Table 2 and Table S2). *GRIN2A* had a significantly increased burden of VEPHMI variation in the EOP cohort (*p* = 0.004). Seven individuals with EOP were carriers of a rare VEPHMI *GRIN2A* variant (20% of the cohort, see Table 3). No VEPHMI rare variants in *GRIN2A* were identified in the first comparison cohort. Among EOP participants, the average age of symptom onset for VEPHMI variant carriers (9.9 years, SD: 2.8) (Table 4) was very similar to the average age of onset for the entire cohort. *GRIN2A* was the only *SCHEMA* gene that showed a statistically significant increased burden in the EOP group relative to the controls after controlling for multiple comparisons. However, the burden of VEPHMI variants in *SRCAP* genes was nominally increased in the EOP group (*p* = 0.04). 

Additional Control Analyses: To confirm the finding, *GRIN2A* variants were compared against three additional race-matched control cohorts (N = 34 each) using SKAT. A significantly increased burden of *GRIN2A* VEPHMI variation was found in the EOP cohort compared to two of the additional control cohorts (*p* = 0.02 for both). An increased burden of *GRIN2A* in the EOP cohort was trending towards significance in comparison to the third additional control cohort (*p* = 0.06) (Supplementary Tables S3 and S4). Analysis of a set of 138 housekeeping genes revealed no significant differences in variant burden between cases and all four sets of controls. 

Interpretation: While the identified *GRIN2A* VEPHMI variants were low allele frequency and missense or splice site variants, they were all predicted to be benign with the exception of one variant of unknown significance (VUS, chr16-9934641 G>T, p.Ala505Glu) using the American College of Medical Genetics (ACMG) criteria (Table 5). The individual with EOP and this variant has a diagnosis of EOP with co-occurring epilepsy and macrocephaly. The variant is inherited from the individual’s mother who does not have seizures or epilepsy but has a diagnosis of ADHD (Table 6). The Combined Annotation Dependent Depletion (CADD) score for this variant was 25, indicating it was one of the top 1% of deleterious variants in the human genome [32].

Variants were inherited in all individuals where it was possible to be assessed (N = 4; see Table 6). Positive family histories of neuropsychiatric disease were noted in all families. In particular, out of four *GRIN2A* variant carrier parents, three had a history of neuropsychiatric disease.

## 4. Discussion

Despite a relatively small sample size, *GRIN2A* rare variant burden was increased in our cohort of individuals with EOP in comparison to a total of four control cohorts. This finding is in accordance with past research that identified an association between *GRIN2A* common polymorphisms (rs7206256 and rs11644461) and childhood-onset schizophrenia [33]. While the variants are not pathogenic according to ACMG criteria, and we are not able to confirm the impact, if any, of these variants on the psychosis pathology of the children and adolescents in the EOP cohort, our findings provide additional support for a role of *GRIN2A* in EOP and neuropsychiatric disease and represent a partial replication of the SCHEMA consortium. 

Multiple lines of evidence suggest that the Glutamate Ionotropic Receptor N-methyl-D-aspartate (NMDA) Type Subunit 2A (*GRIN2A*) gene plays an important role in the pathobiology of psychotic disorders. In addition to the SCHEMA study [14], rare de novo *GRIN2A* mutations are believed to contribute to sporadic cases of adult-onset schizophrenia [34], and common variation in *GRIN2A* (polymorphisms rs7206256 and rs11644461) appears to influence the risk of childhood-onset schizophrenia [33]. These findings generally support the glutamatergic hypothesis of psychosis [35], which postulates that glutamatergic hypofunction in NMDA receptors is a mechanism of disease [36,37,38]. 

Located at 16p13.2, *GRIN2A* is a GluN2A subunit in the NMDA receptor. NMDA receptors are reliant on the binding of D-Serine to the GluN1 subunit and the binding of Glutamate to the GluN2 subunit [39]. NMDA receptor hypofunction is associated with memory and learning impairments as well as psychosis [40]. Mouse models have determined that dysfunction of NMDA receptors influences symptoms associated with schizophrenia, such as anxiety and memory loss [41]. Further, knockout mouse models of *GRIN2A* show similarities to schizophrenia models [42]. The rat model has shown that *GRIN2A* expression is altered in the prefrontal cortex during earlier stages of development, which could impact symptom severity [43]. Literature associating *GRIN2A* common polymorphisms with schizophrenia implies that more mild changes in expression also contribute to neuropsychiatric disorder etiology [33,44,45].

*GRIN2A* is a highly sequence-constrained gene, with a pLi of 1 and a loss-of-function observed/expected upper bound fraction (LOEUF) score of 0.188 (GnomAD, accessed 1 December 2022) [46]. Five of the seven *GRIN2A* variants had a CADD score of 20 or above, indicating they were amongst the top 1% of deleterious variants in the human genome [32]. While the ACMG scoring of these variants was unimpressive with only one reaching the criteria of VUS, this is to be expected for variants with low penetrance that influence the risk for common disease, as these guidelines were not developed for the interpretation of variants in genes associated with complex disorders. While we and others have identified Mendelian forms of EOP [7,13,24], it is clear that not all cases appear to be Mendelian in nature. It is possible that these variants are contributing to a background risk of neuropsychiatric disorders and additional genetic burden is necessary for the EOP phenotype to occur. After all, all individuals with EOP in our cohort had a family history of neuropsychiatric disorders, such as bipolar disorder, depression, and ADHD. In EOP cohort participants 2 and 5, the father was the carrier of the *GRIN2A* variant, but the maternal side had a history of schizophrenia. The interpretation of the functional impact of these variants is unclear.

Our study has several strengths, such as the use of a unique cohort of individuals with EOP and a range of co-occurring conditions, which is highly representative of children and adolescents with psychotic spectrum disorders. Further, while our EOP sample was small, we were still able to gain important new insight into the potential role that a *SCHEMA* gene plays in the etiopathology of EOP. Nonetheless, it will be important to replicate this finding in additional, larger cohorts.

One limitation of this study is the utilization of parents of children with Mendelian disorders as a comparison cohort, as these individuals may themselves have an increased burden of damaging variants. However, the inclusion of these participants would arguably bias the analysis against significance. Additionally, we do not know the functional impact of any of the *GRIN2A* variants, and, while matched on race, subpopulations can have extremely varying allele frequencies. For example, the frequency of rs7193290 is elevated in some African populations. It is possible that the statistical effect that we found is due to, or impacted by, racial subpopulation frequencies.

Relatively high impact genes such as *GRIN2A* are promising targets for elucidating disease mechanisms. As common polymorphisms of *GRIN2A* have also been found to be associated with early-onset schizophrenia, adult-onset schizophrenia, and schizotypy [33,44,45], investigations into the role of *GRIN2A* in early-onset psychotic spectrum disorders deserves further study. Since EOP diagnoses may shift as affected children and adolescents develop [9,10], it will be especially important for future research to follow this cohort and report on the correlation of *GRIN2A* with different psychosis spectrum phenotypes over time. Continuing to understand the impact of rare *GRIN2A* variants may lead to faster diagnostics and create opportunities to develop tailored therapeutics for individuals with EOP and other complex neuropsychiatric conditions.

## Figures and Tables

**Table 1:** Demographics of the early-onset psychosis cohort (N = 34).

	N (%)
Natal Sex	
Female	13 (38.2)
Male	21 (61.8)
Race	
African American/African	6 (17.6)
Asian or Pacific Islander	1 (2.9)
European American/European	21 (61.8)
Other	1 (2.9)
Declined to answer/unknown	5 (14.7)
Ethnicity	
Hispanic/Latino	7 (20.6)
Non-Hispanic/Latino	20 (58.8)
Other	2 (5.9)
Declined to answer/unknown	5 (14.7)
Current age (years)	
13–18	15 (44.1)
19–25	16 (47.1)
26+	3 (8.8)
Average current age	20 (SD: 4.4)
Age of onset of psychotic symptoms (years)	
<8	10 (29.4)
8–12	18 (52.9)
13–18	6 (17.6)
Average age of onset of psychotic symptoms	9.6 (SD: 3.1)
Co-occurring diagnoses	
Anxiety	12 (35.3)
ADHD	18 (52.9)
ASD	9 (26.5)
Depression	12 (35.3)
History of developmental delays	11 (32.4)
History of epilepsy or seizures	11 (32.4)
Intellectual disability	5 (14.7)

Note: ASD = autism spectrum disorder; ADHD = attention-deficit/hyperactivity disorder.

**Table 2:** SKAT analysis of *SCHEMA* genes (*p* = 0.005 is significant after Bonferroni correction).

Chrom	Bp Start	Bp Stop	Gene Symbol	Number of Markers	*p*-Value	Number of Markers Tested
chr1	161016735	161039760	*ARHGAP30*	7	0.49585008	7
chr1	231297857	231357302	*TRIM67*	4	0.63673534	2
chr16	9852375	10276611	*GRIN2A*	11	0.00367797	5
chr16	30709529	30755602	*SRCAP*	11	0.04454448	8
chr17	17584786	17714767	*RAI1*	16	0.3747328	5
chr19	47222763	47250251	*STRN4*	2	NA	NA
chr21	45958863	45960078	*KRTAP10-1*	6	0.8630309	4
chr4	151185593	151936879	*LRBA*	14	0.35271382	10
chr6	139561197	139613276	*TXLNB*	5	0.24115499	4
chr7	150709296	150721586	*ATG9B*	11	0.10667966	8

**Table 3:** Identified VEPHMI *GRIN2A* variants and allele frequencies.

Chrom	POS	REF	Alt	Max Consequence	Carrier Count	Allele Freq	*p*-Value Fisher	Ref Case Count	Het Case Count	Hom Case Count	Ref Ctrl Count	Het Ctrl Count	Hom Ctrl Count
chr16	9858054	T	TCGG	Protein altering variant	0	0.0044	1	34	0	0	31	0	0
chr16	9858055	T	TG	Frameshift variant	0	0.0045	1	34	0	0	31	0	0
chr16	9858072	G	C	Stop gained	0	0.0044	1	34	0	0	31	0	0
chr16	9858074	TTTGG	T	Frameshift variant	0	0.0044	1	34	0	0	31	0	0
chr16	9858079	T	TAAAAAA	Inframe insertion	0	0.0045	1	34	0	0	31	0	0
chr16	9858173	G	T	Missense variant	2	0.0168	0.25	32	2	0	34	0	0
chr16	9934641	G	T	Missense variant	1	0.0042	1	33	1	0	33	0	0
chr16	9934969	G	A	Splice region variant	1	0.0042	1	33	1	0	33	0	0
chr16	9943800	G	A	Missense variant	0	0.0042	1	34	0	0	34	0	0
chr16	10031844	G	C	Missense variant	2	0.0085	0.5	32	2	0	32	0	0
chr16	10032161	T	C	Missense variant	1	0.0042	1	33	1	0	34	0	0

**Table 4:** Demographics of *GRIN2A* Carriers.

ID	Natal Sex	Race	Ethnicity	Age of Onset of Psychotic Symptoms
1	Male	European American/European	Non-Hispanic	9
2	Male	European American/European	Non-Hispanic	12
3	Male	European American/European	Non-Hispanic	12
4	Female	African American/African	Hispanic	10
5	Male	European American/European	Hispanic	9
6	Female	European American/European	Non-Hispanic	4
7	Male	African American/African	Declined to answer	13

**Table 5:** ACMG Interpretations of amino acid changes identified in individuals with early-onset psychosis.

Chrom	POS (GRCh37)	REF	Alt	Amino Acid Change	Het OR Hom	ACMG Interpretation	Categories	Rs Number
chr16	9858173	G	T	p.Asn1076Lys	het	Benign	BA1, BS2, BP4, BP6	rs61758995
chr16	9858173	G	T	p.Asn1076Lys	het	Benign	BA1, BS2, BP4, BP6	rs61758995
chr16	9934641	G	T	p.Ala505Glu	het	VUS	PM2, PM1	
chr16	9934969	G	A		het	Benign	BA1, BS2, BP4, BP6	rs7193290
chr16	10031844	G	C	p.Pro327Ala	het	Likely Benign	PM2, BS2, BP4	rs771168389
chr16	10031844	G	C	p.Pro327Ala	het	Likely Benign	PM2, BS2, BP4	rs771168389
chr16	10032161	T	C	p.Lys221Arg	het	Benign	BA1, BS2, BP6	rs61731464

**Table 6:** Diagnoses, co-occurring conditions, inheritance, and family phenotypes of individuals with *GRIN2A* variants in the early-onset psychosis cohort.

ID	Diagnosis	Co-Occurring Condition(s)	Inheritance	Carrier Parent Phenotype	Other Family History of Neuropsychiatric Disease
1	Early-onset schizophrenia	ASD	Paternal	Anxiety, depression, substance use disorder	None noted
2	Early-onset psychosis	OCD	Paternal	None noted	Maternal uncle: schizophrenia
3	Early-onset psychosis	Epilepsy, microcephaly	Maternal	ADHD	Brother: seizures Family history of bipolar disorder
4	Early-onset psychosis	Adenoidectomy, ADHD combined type, asthma, bipolar II disorder, obesity, type 2 diabetes mellitus	Unknown	Unknown	Mother: ADHD Father: PTSD and substance use Maternal grandmother: depression
5	Early-onset psychosis		Paternal	Severe OCD without psychotic symptoms	Maternal family history of bipolar disorder, schizophrenia, and ADHD
6	Early-onset psychosis	ADHD, ASD, Chiari malformation, seizures	Unknown	Unknown	Maternal grandmother: bipolar disorder
7	Early-onset psychosis	Depression, PTSD, reactive attachment disorder	Unknown	Unknown	Mother: seizures Father: childhood epilepsy (now outgrown)

Note: ASD = autism spectrum disorder; OCD = obsessive compulsive disorder; ADHD = attention-deficit/hyperactivity disorder; PTSD = post-traumatic stress disorder.

## Data Availability

The data presented in this study are available on request from the corresponding author. The data are not publicly available due to privacy concerns.

## Supplementary Materials

The following supporting information can be downloaded at: https://www.mdpi.com/article/10.3390/genes14040779/s1, Table S1: Housekeeping gene list; Table S2: SKAT markers and allele frequencies of 10 *SCHEMA* genes; Table S3: Repeated SKAT *GRIN2A* analysis of cases vs. 3 control cohorts; Table S4: SKAT markers and allele frequencies of *GRIN2A* variants in cases vs. 3 control cohorts.
