# Supplementary material for: Children with Early-Onset Psychosis Have Increased Burden of Rare GRIN2A Variants
Source: Genes (Basel). 2023 Mar 23;14(4):779. doi: 10.3390/genes14040779 (PMC10138040; doi:10.3390/genes14040779)
Supplement: Supplementary file 1 [file genes-14-00779-s001.zip › genes-2130936-supplementary.pdf]

Supplementary table S1. Housekeeping gene list.

**MTFR1L**	**PPARA**	**DHX30**	**HNRNPUL2**	**CHMP6**	**RPF2**
**KPNA6**	CHCHD4	PPP4R2	MAP3K11	PSMG2	SYNJ2
**STK40**	SLTM	TMEM39A	PAAF1	MIB1	CHST12
**RNF220**	FAM96A	KPNA1	PANX1	RPL17	CXorf40A
**TESK2**	TRIP4	SLC9A9	FAM76B	CC2D1A	IFRD1
**TMEM59**	MORF4L1	SLC33A1	ATP5L	SLC35E1	LUZP6
**AHCYL1**	SNRNP25	KPNA4	EI24	TBCB	ADCK2
**AP4B1**	DNAJA3	ZNF75D	PRDM10	ZNF347	FAM160B2
**TRIM33**	DCTN5	RUFY3	WNK1	RDH13	TGS1
**APH1A**	ZNF668	FNIP2	C12orf4	PPP6R1	LACTB2
**TOMM40L**	TK2	FASTKD3	MRPS35	KIDINS220	EIF3E
**C1orf27**	DDX19B	ERCC8	C12orf73	PREB	TRAPPC9
**SMYD3**	NAA10	AGGF1	ANAPC7	PPM1G	TSTA3
**HSPA14**	CLUH	CHD1	UNC119B	TTC27	NDUFB6
**PDSS1**	CTDNEP1	ATG12	RNF34	CRIPT	ISCA1
**DNAJB12**	MPDU1	KDM3B	RNASEH2B	FARSB	IARS
**MRPS16**	PER1	HARS	MYCBP2	WDFY1	DENND1A
**POLR3A**	RANGRF	ANXA6	TFDP1	SNRPB	PRRC2B
**CCNJ**	AATF	SFXN1	NEDD8	CTSA	NACC2
**HPS1**	MED1	NSD1	SNX6	GART	INPP5E
**DNMBP**	RAB5C	MGAT4B	PSEN1	C22orf39	ACOT9
**DCLRE1A**	GHDC	RANBP9	YLPM1	TXNRD2	ELK1
**RTF1**	SPOP	PFDN6	DTWD1	SGSM3	GRIPAP1
**FBXO3**	MAP3K3	TMEM30A	INO80	POLR3H	STAG2

Supplementary Table S2. SKAT markers and allele frequencies of 10 SCHEMA genes.

Chrom	POS	REF	alt	gene symbol	max consequence	carrier count	Allele freq	pVal Fisher	ref case count	het case count	hom case count	ref ctrl count	het ctrl count	hom ctrl count
chr1	161017761	C	T	ARHGAP30	missense variant	4	0.02101	1	32	2	0	32	2	0
chr1	161018224	C	T	ARHGAP30	missense variant	1	0.00424	1	33	0	0	33	1	0
chr1	161018268	T	C	ARHGAP30	missense variant	2	0.0084	0.25	32	2	0	34	0	0
chr1	161018374	C	T	ARHGAP30	missense variant	1	0.0042	1	34	0	0	33	1	0
chr1	161018824	C	T	ARHGAP30	missense variant	1	0.0042	1	33	1	0	34	0	0
chr1	161018896	C	T	ARHGAP30	missense variant	1	0.01681	1	33	1	0	34	0	0
chr1	161023074	T	C	ARHGAP30	missense variant	1	0.01271	1	33	1	0	33	0	0
chr1	231299470	A	AGCC	TRIM67	inframe insertion	0	0.0045	1	32	0	0	28	0	0
chr1	231299470	AGCC	A	TRIM67	inframe deletion	2	0.00909	0.5	30	2	0	27	0	0
chr1	231299533	G	A	TRIM67	missense variant	0	0.0045	1	32	0	0	28	0	0
chr1	231344908	A	G	TRIM67	missense variant	2	0.0084	1	33	1	0	33	1	0
chr16	9858054	T	TCGG	GRIN2A	protein altering variant	0	0.00446	1	34	0	0	31	0	0
chr16	9858055	T	TG	GRIN2A	frameshift variant	0	0.0045	1	34	0	0	31	0	0
chr16	9858072	G	C	GRIN2A	stop gained	0	0.00446	1	34	0	0	31	0	0
chr16	9858074	TTTGG	T	GRIN2A	frameshift variant	0	0.00446	1	34	0	0	31	0	0
chr16	9858079	T	TAAAAAA	GRIN2A	inframe insertion	0	0.0045	1	34	0	0	31	0	0
chr16	9858173	G	T	GRIN2A	missense variant	2	0.01681	0.25	32	2	0	34	0	0
chr16	9934641	G	T	GRIN2A	missense variant	1	0.00424	1	33	1	0	33	0	0
chr16	9934969	G	A	GRIN2A	splice region variant	1	0.00424	1	33	1	0	33	0	0
chr16	9943800	G	A	GRIN2A	missense variant	0	0.0042	1	34	0	0	34	0	0
chr16	10031844	G	C	GRIN2A	missense variant	2	0.00855	0.5	32	2	0	32	0	0
chr16	10032161	T	C	GRIN2A	missense variant	1	0.0042	1	33	1	0	34	0	0
chr16	30723148	C	T	SRCAP	splice region variant	1	0.0042	1	33	1	0	34	0	0
chr16	30732143	T	C	SRCAP	missense variant	1	0.0042	1	33	1	0	34	0	0
chr16	30732558	C	A	SRCAP	missense variant	0	0.0042	1	34	0	0	34	0	0
chr16	30732644	C	T	SRCAP	missense variant	1	0.0042	1	33	1	0	34	0	0

chr16	30734337	G	C	SRCAP	missense variant	1	0.0042	1	33	1	0	34	0	0
chr16	30735348	C	G	SRCAP	missense variant	1	0.01709	0.48	31	1	0	34	0	0
chr16	30736263	C	T	SRCAP	missense variant	1	0.0042	1	33	1	0	34	0	0
chr16	30736370	G	GC	SRCAP	frameshift variant	1	0.0042	1	33	1	0	34	0	0
chr16	30740333	A	G	SRCAP	missense variant	1	0.0042	1	34	0	0	33	1	0
chr16	30750063	T	C	SRCAP	missense variant	0	0.0042	1	34	0	0	34	0	0
chr16	30750614	A	G	SRCAP	missense variant	0	0.0042	1	34	0	0	34	0	0
chr17	17696987	C	T	RAI1	missense variant	0	0.00917	1	24	0	0	34	0	0
chr17	17697093	CCAGCAGCAG	C	RAI1	inframe deletion	1	0.00495	0.44	21	1	0	28	0	0
chr17	17697093	CCAGCAGCAGCAG	C	RAI1	inframe deletion	1	0.02475	1	22	0	0	27	1	0
chr17	17697093	CCAGCAGCAGCAGCAGCAGCAG	C	RAI1	inframe deletion	2	0.0099	0.5	22	0	0	26	2	0
chr17	17697093	C	CCAG	RAI1	inframe insertion	0	0.01485	1	22	0	0	28	0	0
chr17	17697093	CCAGCAG	C	RAI1	inframe deletion	1	0.00495	0.44	21	1	0	28	0	0
chr17	17697101	AGCAG	A	RAI1	frameshift variant	1	0.00521	0.45	20	1	0	26	0	0
chr17	17697101	AGCAGCAGCAG	A	RAI1	frameshift variant	1	0.005	0.43	20	1	0	28	0	0
chr17	17697404	C	T	RAI1	missense variant	3	0.01681	0.62	32	2	0	33	1	0
chr17	17698006	G	A	RAI1	missense variant	1	0.0042	1	33	1	0	34	0	0
chr17	17698178	C	T	RAI1	missense variant	1	0.0042	1	34	0	0	33	1	0
chr17	17699141	G	A	RAI1	missense variant	0	0.0042	1	34	0	0	34	0	0
chr17	17699252	G	A	RAI1	missense variant	0	0.0042	1	34	0	0	34	0	0
chr17	17699992	CGCA	C	RAI1	inframe deletion	1	0.0042	1	33	1	0	34	0	0
chr17	17699992	CGCAGCA	C	RAI1	inframe deletion	1	0.0042	1	33	1	0	34	0	0
chr17	17700037	AAGG	A	RAI1	inframe deletion	0	0.0042	1	34	0	0	34	0	0
chr19	47223986	G	A	STRN4	missense variant	2	0.0084	0.25	32	2	0	34	0	0
chr19	47226434	C	T	STRN4	missense variant	4	0.01695	1	31	2	0	32	2	0
chr21	45959256	C	G	KRTAP10-1	missense variant	0	0.0084	1	34	0	0	34	0	0
chr21	45959279	C	T	KRTAP10-1	missense variant	1	0.0042	1	33	1	0	34	0	0
chr21	45959292	CA	TG	KRTAP10-1	missense variant	1	0.0042	1	34	0	0	33	1	0
chr21	45959945	T	G	KRTAP10-1	missense variant	0	0.0084	1	34	0	0	34	0	0

chr21	45959953	ACAG	A	KRTAP10-1	inframe deletion	1	0.0042	1	33	1	0	34	0	0
chr21	45959972	G	T	KRTAP10-1	missense variant	3	0.01681	1	33	1	0	32	2	0
chr4	151207098	C	G	LRBA	missense variant	1	0.0042	1	33	1	0	34	0	0
chr4	151207180	G	A	LRBA	missense variant	0	0.0042	1	34	0	0	34	0	0
chr4	151236754	T	G	LRBA	missense variant	3	0.01282	0.24	31	3	0	32	0	0
chr4	151242409	T	G	LRBA	missense variant	1	0.0084	1	34	0	0	33	1	0
chr4	151336707	C	T	LRBA	missense variant	1	0.0042	1	33	1	0	34	0	0
chr4	151509335	A	C	LRBA	splice region variant	1	0.00435	1	33	1	0	30	0	0
chr4	151520216	G	A	LRBA	missense variant	4	0.03017	0.35	33	1	0	28	3	0
chr4	151727540	C	A	LRBA	stop gained	1	0.0042	1	34	0	0	33	1	0
chr4	151791682	T	C	LRBA	missense variant	0	0.0042	1	34	0	0	34	0	0
chr4	151793903	T	C	LRBA	missense variant	3	0.02521	1	33	1	0	32	2	0
chr4	151827146	C	A	LRBA	splice region variant	0	0.00431	1	34	0	0	31	0	0
chr4	151829580	T	C	LRBA	missense variant	0	0.0042	1	34	0	0	34	0	0
chr4	151837660	G	C	LRBA	missense variant	1	0.0042	1	34	0	0	33	1	0
chr4	151850224	AGG	A	LRBA	splice region variant	1	0.0042	1	34	0	0	33	1	0
chr6	139563937	G	A	TXLNB	missense variant	3	0.01261	0.24	31	3	0	34	0	0
chr6	139564117	G	C	TXLNB	missense variant	1	0.0042	1	33	1	0	34	0	0
chr6	139569043	T	C	TXLNB	missense variant	0	0.0042	1	34	0	0	34	0	0
chr6	139583914	C	A	TXLNB	splice region variant	2	0.01681	1	33	1	0	33	1	0
chr6	139610006	C	T	TXLNB	missense variant	2	0.01261	1	33	1	0	33	1	0
chr7	150713042	C	T	ATG9B	missense variant	1	0.0042	1	34	0	0	33	1	0
chr7	150713218	TCTC	T	ATG9B	inframe deletion	1	0.0042	1	34	0	0	33	1	0
chr7	150714163	G	A	ATG9B	missense variant	1	0.0042	1	34	0	0	33	1	0
chr7	150715383	C	G	ATG9B	missense variant	0	0.00427	1	34	0	0	33	0	0
chr7	150720997	GCTC	G	ATG9B	inframe deletion	2	0.0084	0.25	32	2	0	34	0	0
chr7	150721014	G	A	ATG9B	missense variant	2	0.0084	0.25	32	2	0	34	0	0

chr7	150721026	G	C	ATG9B	missense variant	1	0.0042	1	34	0	0	33	1	0
chr7	150721057	C	T	ATG9B	missense variant	0	0.0042	1	34	0	0	34	0	0
chr7	150721090	G	A	ATG9B	missense variant	1	0.02101	1	33	1	0	34	0	0
chr7	150721396	AAGG	A	ATG9B	inframe deletion	0	0.00847	1	34	0	0	34	0	0
chr7	150721483	T	TC	ATG9B	frameshift variant	1	0.00442	1	31	1	0	32	0	0

Supplementary Table S3. Repeated SKAT *GRIN2A* analysis of cases vs. three control cohorts.

**Control cohort #**	**Chrom**	**Bp start**	**Bp stop**	**Gene Symbol**	**Num Markers**	**P value**
**Control cohort 2**	**chr16**	**9852375**	**10276611**	**GRIN2A**	**8**	**0.017**
Control cohort 3	**chr16**	**9852375**	**10276611**	**GRIN2A**	**8**	**0.018**
Control cohort 4	**chr16**	**9852375**	**10276611**	**GRIN2A**	**8**	**0.06**

Supplementary Table S4. SKAT markers and allele frequencies of *GRIN2A* variants in cases vs. three control cohorts.

control cohort 2

Chrom	POS	REF	alt	max consequence	carrier count	Allele freq	pVal Fisher	ref case count	het case count	hom case count	ref ctrl count	het ctrl count	hom ctrl count
**Control cohort 2**													
**chr16**	9858173	G	T	missense variant	2	0.013	0.25	32	2	0	34	0	0
**chr16**	9858502	C	G	missense variant	0	0.006	1	34	0	0	34	0	0
**chr16**	9858636	G	A	missense variant	1	0.003	1	34	0	0	33	1	0
**chr16**	9934641	G	T	missense variant	1	0.003	1	33	1	0	34	0	0
**chr16**	9934969	G	A	splice region variant	1	0.006	1	33	1	0	34	0	0
**chr16**	10031844	G	C	missense variant	2	0.006	0.25	32	2	0	34	0	0
**chr16**	10032161	T	C	missense variant	1	0.003	1	33	1	0	34	0	0
**chr16**	10274192	G	A	missense variant	0	0.004	1	27	0	0	34	0	0

Control cohort 3

Chrom	POS	REF	alt	max consequence	carrier count	Allele freq	pVal Fisher	ref case count	het case count	hom case count	ref ctrl count	het ctrl count	hom ctrl count
**chr16**	9858173	G	T	missense variant	2	0.013	0.25	32	2	0	34	0	0
**chr16**	9858502	C	G	missense variant	1	0.006	1	34	0	0	33	1	0
**chr16**	9858636	G	A	missense variant	0	0.003	1	34	0	0	34	0	0
**chr16**	9934641	G	T	missense variant	1	0.003	1	33	1	0	34	0	0
**chr16**	9934969	G	A	splice region variant	1	0.006	1	33	1	0	33	0	0
**chr16**	10031844	G	C	missense variant	2	0.006	0.25	32	2	0	34	0	0
**chr16**	10032161	T	C	missense variant	1	0.003	1	33	1	0	34	0	0
**chr16**	10274192	G	A	missense variant	0	0.004	1	27	0	0	26	0	0

Control cohort 4

Chrom	POS	REF	alt	max consequence	carrier count	Allele freq	pVal Fisher	ref case count	het case count	hom case count	ref ctrl count	het ctrl count	hom ctrl count
**chr16**	9858173	G	T	missense variant	4	0.013	1	32	2	0	31	2	0
**chr16**	9858502	C	G	missense variant	0	0.006	1	34	0	0	34	0	0
**chr16**	9858636	G	A	missense variant	0	0.003	1	34	0	0	33	0	0
**chr16**	9934641	G	T	missense variant	1	0.003	1	33	1	0	31	0	0
**chr16**	9934969	G	A	splice region variant	1	0.006	1	33	1	0	33	0	0
**chr16**	10031844	G	C	missense variant	2	0.006	0.5	32	2	0	31	0	0
**chr16**	10032161	T	C	missense variant	1	0.003	1	33	1	0	33	0	0
**chr16**	10274192	G	A	missense variant	0	0.004	1	27	0	0	26	0	0
